# Social Distance during the COVID-19 Pandemic Reflects Perceived Rather Than Actual Risk

**DOI:** 10.3390/ijerph18115504

**Published:** 2021-05-21

**Authors:** Tina Iachini, Francesca Frassinetti, Francesco Ruotolo, Filomena Leonela Sbordone, Antonella Ferrara, Maria Arioli, Francesca Pazzaglia, Andrea Bosco, Michela Candini, Antonella Lopez, Alessandro Oronzo Caffò, Zaira Cattaneo, Ferdinando Fornara, Gennaro Ruggiero

**Affiliations:** 1 Department of Psychology, Università degli Studi della Campania “L. Vanvitelli”, 81100 Caserta, Italy; santa.iachini@unicampania.it (T.I.); filomenaleonela.sbordone@unicampania.it (F.L.S.); antonella.ferrara@unicampania.it (A.F.); gennaro.ruggiero@unicampania.it (G.R.); 2 Department of Psychology, Università di Bologna, 40127 Bologna, Italy; francesc.frassinetti@unibo.it (F.F.); michela.candini2@unibo.it (M.C.); 3 Istituti Clinici Scientifici Maugeri-IRCCS di Castel Goffredo, Castel Goffredo, 46042 Mantova, Italy; 4 Department of Psychology, Università degli Studi di Milano-Bicocca, 20126 Milano, Italy; maria.arioli@unimib.it (M.A.); zaira.cattaneo@unimib.it (Z.C.); 5 Department of General Psychology, Università degli Studi di Padova, 35121 Padova, Italy; francesca.pazzaglia@unipd.it; 6 Department of Educational Sciences, Psychology, Communication, Università degli Studi di Bari Aldo Moro, 70121 Bari, Italy; andrea.bosco@uniba.it (A.B.); antonella.lopez@uniba.it (A.L.); alessandro.caffo@uniba.it (A.O.C.); 7 IRCCS Mondino Foundation, 27100 Pavia, Italy; 8 Department of Education, Psychology, Philosophy, Università di Cagliari, 09123 Cagliari, Italy; ffornara@unica.it

**Keywords:** social distancing, interpersonal space, COVID-19, risk perception, anxiety

## Abstract

Interpersonal space (IPS) is the area surrounding our own bodies in which we interact comfortably with other individuals. During the COVID-19 pandemic, keeping larger IPS than usual, along with wearing a face mask, is one of the most effective measures to slow down the COVID-19 outbreak. Here, we explore the contribution of actual and perceived risk of contagion and anxiety levels in regulating our preferred social distance from other people during the first wave of the COVID-19 pandemic in Italy. In this study, 1293 individuals from six Italian regions with different levels of actual risk of infection participated in an online survey assessing their perceived risk to be infected, level of anxiety and IPS. Two tasks were adopted as measures of interpersonal distance: the Interpersonal Visual Analogue Scale and a questionnaire evaluating interpersonal distance with and without face mask. The results showed that the IPS regulation was affected by how people subjectively perceived COVID-19 risk and the related level of anxiety, not by actual objective risk. This clarifies that the role of threat in prompting avoidant behaviors expressed in increased IPS does not merely reflect environmental events but rather how they are subjectively experienced and represented.

## 1. Introduction

Interpersonal space (IPS) is the area surrounding our own bodies in which we interact with other individuals [1]. This portion of space has been defined as an emotionally tinged area that an individual feels is private space, a space where any unwanted intrusion by others causes discomfort, anger or fear [1,2,3]. Typically, individuals regulate IPS through two basic behaviors: they extend their distance when they feel they are in dangerous and uncomfortable situations (avoidance behavior), and vice-versa, they reduce their distance when they feel they are in friendly and safe situations (approach behavior) [1,3,4,5,6].

According to Hall’s classic model [1], distance during more formal interactions is larger than distance during more intimate interactions. Moreover, the proxemics literature has shown that IPS can be modulated by individual differences and situational factors (for reviews, [2,7,8,9]). For example, IPS is usually larger from males than females, and it increases as the age of the interactants increases [2,10,11,12,13,14,15]. In addition, psychological factors such as anxiety affect the size of the IPS (i.e., the higher the anxiety levels the larger the IPS, see [16,17,18,19]; for a review, [2]). Interestingly for the aim of the present study, IPS is larger when people feel a high perceived risk in the presence of novel threats that cannot be controlled [20,21,22,23,24].

In this regard, a very relevant situation is represented by the COVID-19 pandemic that spread worldwide in 2020 and is still affecting millions of people in 2021. In this situation, keeping larger-than-usual interpersonal social distances, along with wearing a face mask, are some of the most effective measures to slow down the COVID-19 outbreak [25,26] (for a review, [27]). Even if the situation is improving nowadays thanks to the possibility of vaccination, maintaining large interpersonal distances and wearing face masks is still highly recommended for controlling the COVID-19 outbreak. As yet, we do not have enough vaccine to protect everyone, and we do not know the extent to which vaccinated people continue to be at risk of infection and transmission of infection to others [28].

Therefore, it is important to understand which factors drive individuals’ regulation of interpersonal distances. In this study, we asked how the actual severity of contagion, perceived risk of contagion and anxiety levels may have affected preferred social distance from other people.

To reach our aims, we studied the regulation of IPS during the first wave of the COVID-19 pandemic in Italy, when the whole country was submitted to a period of severe lockdown (from 9 March to 4 May 2020, Decree of the Prime Minister, 9 March 2020). In those days, there was a social climate of anxiety, concern and awareness of the need for confinement. The Italian case is particularly interesting because in that period the severity of the pandemic changed substantially depending on the territorial area. We analyzed seven dimensions that contributed to defining the actual severity of the infection or actual risk (i.e., number of positive subjects, new positive cases, increase of total cases compared with previous day, number of patients in intensive care, number of all hospital discharges, number of deaths, number of swabs) (Istituto Superiore di Sanità—ISS 2020). According to these criteria, we chose six regions that reflected different actual risk levels: very high (Lombardy), medium-high (Emilia-Romagna and Veneto), medium-low (Campania and Apulia), very low (Sardinia).

In the current study, we recruited a sample of female and male participants (*N* = 1293) of different ages living there. All participants completed an online survey in which perceived risk of contagion, level of anxiety (State-Trait Anxiety Inventory, STAI, [29]) and interpersonal space were assessed. An ad hoc questionnaire was used to evaluate the perceived risk, in the present and in the future, taking into account the level of risk in the area in which the participant lived (local risk) and the overall risk (general risk). Two tasks were adopted as a measure of interpersonal distance: the Interpersonal Visual Analogue Scale (IVAS [12]) and a questionnaire to measure interpersonal distance with and without a face mask. In the IVAS, two characters were presented on opposite sides of a line, and participants were required to indicate, by moving a slider, their preferred interpersonal distance. This task has already been used in a previous study [12] that revealed comparable results with behavioral studies, showing effects of gender and age in the regulation of interpersonal distances.

Two possible scenarios can be expected. If interpersonal distance during the pandemic were mainly driven by actual risk, then an effect of the region where participants lived should be found: the higher the actual risk, the larger the IPS. Conversely, if perceived risk had a main role in defining the preferred IPS, then we would expect that the higher the perceived risk, the larger the IPS, regardless of the actual risk. Since wearing a face mask reduces the risk of infection, we would expect a reduction in IPS with than without a face mask. Furthermore, we hypothesized that anxiety levels should also affect IPS, i.e., higher anxiety levels should increase the IPS. Finally, considering that the COVID-19 risk and its negative outcomes were worse for older adults than younger adults, and especially for males than females (Surveillance Group—ISS 2020), we also expected a larger IPS for males and older adults than for females and younger adults.

## 2. Materials and Methods

### 2.1. Participants

The survey was conducted over 10 days (23 April–2 May 2020) during the lockdown. A total of 1293 participants (F = 854, M = 439) aged 18 to 82 years (M = 35.53; SD = 16.05) took part in the study. Since 142 respondents did not complete the survey, they were excluded from the final sample. The survey required demographic data (i.e., age, sex, level of education, type of employment, province and region of residence). Six Italian universities collaborated in the data collection (i.e., University of Bologna, University of Bari, University of Cagliari, University of Campania, University of Study Milano-Bicocca, University of Padova). As regards sample composition, the number of participants by each region was the following: Lombardy = 218, Emilia-Romagna = 211, Veneto = 184, Campania = 314, Apulia = 210, Sardinia = 156. To verify that our sample size was adequately powered to produce reliable and replicable findings, we conducted a post hoc power analysis with G* Power 3.1 [30]. The analysis was calculated with the following parameters: alpha = 0.05, *N* = 1293 and *f* = 0.11, resulting in a power = 0.8.

Informed consent was obtained from all participants. Recruitment and testing were in accordance with the ethical standards of the Institutional Review Board of the Department of Psychology (University of Campania; N.8 prot. # 16.20) and with the Declaration of Helsinki (2013).

### 2.2. Materials

PsyToolkit was used for the online survey [31,32]. The measures described below were inserted in the survey (because this study was part of a larger research, the survey included a set of other measures that were not the focus of this study).

#### 2.2.1. Measures of IPS

Interpersonal distance was measured by using two tasks: the Interpersonal Visual Analogue Scale (IVAS, [12]) and an ad hoc questionnaire to measure interpersonal distance with or without a face mask.

**Figure 1:**
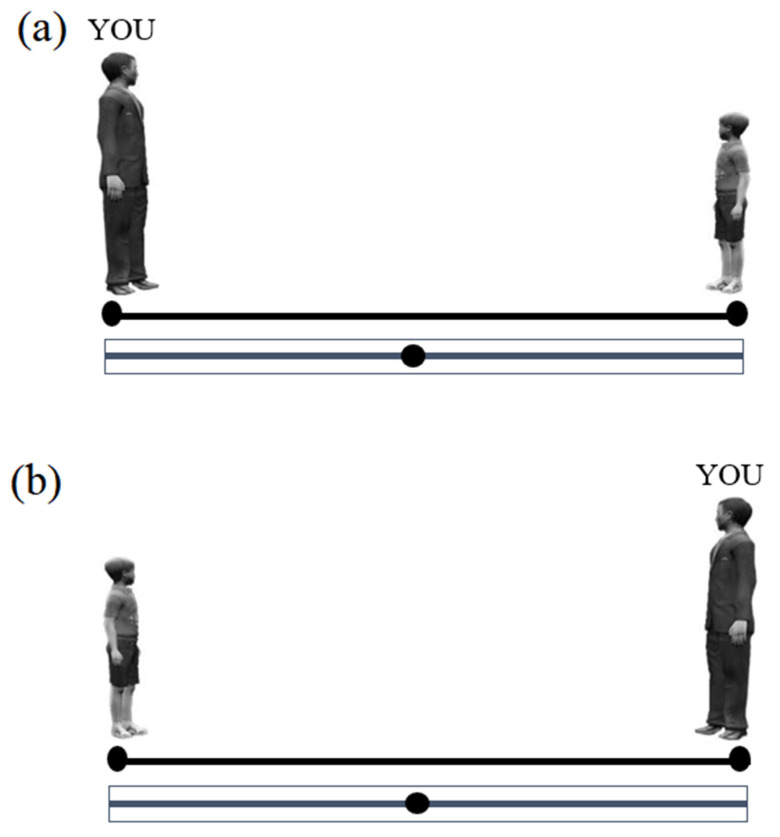
An example of Interpersonal Visual Analogue Scale trials is depicted. The label “YOU” indicates a male participant; the other character on the opposite of the line represents the confederate, i.e., the person with respect to whom the preferred distance is determined (in this case a male child). The preferred distance was to be indicated by moving the slider along the straight gray line: the further the slider was placed from the participant (YOU), the larger the distance from the other person. The character “YOU” could appear in 50% of the trials on the left side (**a**) and on the right side in the other 50% (**b**).

IVAS. A modified on-line version of the IVAS was used [12]. Participants were presented, on a PC screen, with images depicting two different characters on opposite sides of a line: one character (male or female) for the participant (self), the other for another person (confederate). Six different confederates were presented (one for each trial): a child (about 8–10 years old, height = 24 mm), a young adult (about 30 years old, height = 32 mm) or an elderly adult (about 65–70 years old, height = 32 mm), male or female. The initial distance between the self and the confederate was 100 mm. Participants had to imagine the confederate walking toward them and had to indicate, by moving a slider, their preferred interpersonal distance from the confederate (from max 100 to min 0) (see Figure 1).

Face mask metric questionnaire. Participants had to indicate the distance from others that made them feel comfortable among eight possible alternatives (0.50–1–1.5–2–2.5–3–3.5–4 m) in two conditions: imaging wearing or not wearing a face mask.

#### 2.2.2. Actual Risk

To measure the actual COVID-19 risk, we computed a comprehensive index across the six regions where the respondents lived. The index was calculated considering seven dimensions of infection severity for each day of the data collection period: number of positive subjects, new positive cases, increase of total cases compared with previous day, number of patients in intensive care, number of all hospital discharges (inverted), number of deaths, number of swabs (Source: Istituto Superiore di Sanità, Rome, Italy, 2020). For each region, we obtained an average score across days. The correlations between the seven dimensions were high (between 0.75 and 0.98). To obtain a single composite measure of actual risk, a principal component analysis was conducted on the dimensions. A single-factor solution was extracted (that explained 94% of the variance, with Cronbach’s alpha of 0.98) and a factor score (method: regression) was calculated. Distances expressed by the factor score can be interpreted as *z* scores.

#### 2.2.3. Perceived Risk

Four questions investigated how people perceived the COVID-19 infection risk. The questions concerned: (i) the general perceived risk, i.e., how dangerous did people consider COVID-19 to be, independently of their contingent situation, and (ii) the local perceived risk, i.e., how dangerous did people consider COVID-19 to be in the place where they lived. In both cases, people were asked to relate their beliefs for two times—in the present and in the future: “How dangerous do you consider the coronavirus to be in general in the present/in the future?” and “How dangerous do you consider the coronavirus to be in the area where you live in the present/in the future?” Scores could vary from 100 (max risk) to 0 (no risk).

#### 2.2.4. Anxiety Levels (STAI)

The State-Trait Anxiety Inventory (STAI, [29]; see also [33]) is a self-report inventory based on a 4-point Likert scale. The STAI consists of forty items to measure the state and trait anxiety of participants. The range of scores for each sub-scale is 20–80, the higher score indicating higher anxiety [34,35].

### 2.3. Procedure

After reading the instructions and digitally signing the informed consent, participants started the survey. All participants answered the items and filled in the questionnaires in the following order: (1) demographic information; (2) perceived risk; (3) State Anxiety Inventory; (4) IVAS; (5) face mask metric questionnaire; (6) Trait Anxiety Inventory. The time taken to complete the questionnaire ranged from 15 to 20 min.

### 2.4. Data Analysis

The IVAS task provided the mean distance (mm) chosen by participants with respect to the six types of confederates: female/male child, female/male young adult, female/male elderly adult. The reliability of the six IVAS measures was assessed: Cronbach’s alpha = 0.97, inter-item correlation = 0.87. Considering the strong inter-item correlation and reliability of the six interpersonal distance dimensions, a mean interpersonal distance was calculated and used for regression analyses.

First, to understand the effect of actual and perceived risk and anxiety levels on IPS, a multiple regression analysis was performed, with the mean distance (mm) provided by the IVAS task as criterion and the following predictors: actual risk, the four dimensions of perceived risk (present general, present local, future general, future local), state and trait anxiety (see Table 1 for the separate values in each region).

Second, to assess the role of the confederate’s and participant’s gender and age on IPS, an analysis of covariance (ANCOVA) was performed. Independent variables were: participant’s gender (M/F) as between-subject factor, confederate’s gender (M/F) and confederate’s age (children/young adults/elderly adult) as within-subject factors. Participant age was entered as a covariate. As the dependent variable, we used the distance (in mm) for the six confederates of the IVAS task.

Third, to explore if wearing a face mask influenced social distancing, an analysis of covariance (ANCOVA) was carried out on interpersonal distance obtained by the questionnaire (in meters) with participant’s gender (M/F) as between-subject factor and face mask (with/without) as within-subject factor. Participant’s age was entered as a covariate.

Where necessary, the Bonferroni correction was used for the post hoc tests, and the partial eta-squared (η^2^_p_) is also reported.

## 3. Results

### 3.1. The Effect of Actual/Perceived COVID-19 Risk and Anxiety on IPS

**Figure 2:**
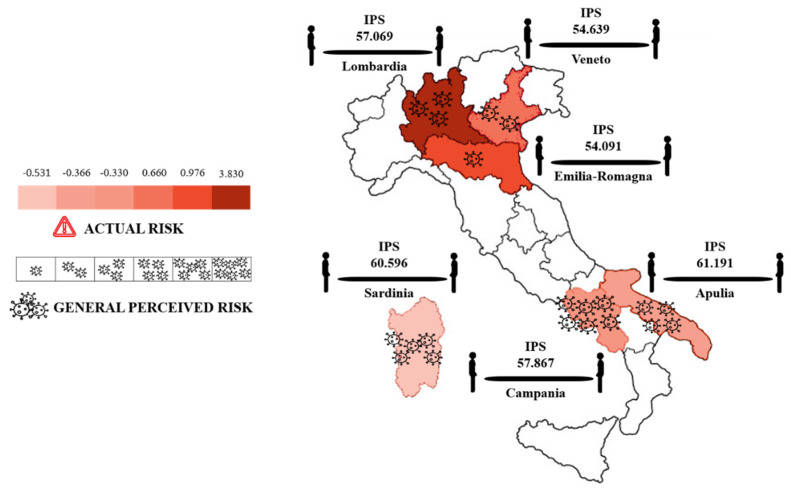
The figure shows the levels of actual risk (darker colors indicate higher levels of COVID-19 actual risk) and present general perceived risk (more virus icons indicate higher levels of perceived risk). Mean interpersonal distance (IPS) measured in the IVAS task for each region is also reported (straight black lines near each region).

The multiple regression analysis revealed that the overall model was significant: *F*(7, 1285) = 16.10, *p* < 0.0001, *R* = 0.28, *R*^2^ = 0.08. As shown in Table 2, the predictors present general perceived risk and state anxiety contributed significantly to the model: the higher the perceived risk of COVID-19 in the present at a general level and the higher the state anxiety, the larger the social distance (see Figure 2).

### 3.2. The Effects of Confederate’s and Participant’s Gender and Age on IPS

**Figure 3:**
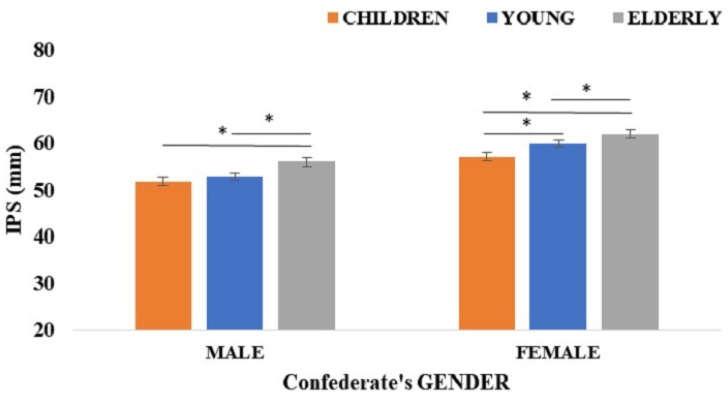
The figure shows the mean IPS (mm) as a function of male and female confederates of different ages (children, young and elderly people). The asterisk indicates a significant (*p* < 0.05) difference between comparisons. Error bars depict standard error of the mean (SEM).

The ANCOVA showed a significant main effect of participant’s gender: *F*(1, 1290) = 28.39, *p* < 0.0001; ƞ^2^_p_ = 0.02, with IPS being larger in female (M = 59.58; SD = 18.20) than male participants (M = 53.53; SD = 16.72). A significant main effect of confederate’s gender also appeared: *F*(1, 1290) = 6.15, *p* < 0.013; ƞ^2^_p_ = 0.01, with larger IPS toward male confederates (M = 57.79; SD = 20.27) than female confederates (M = 57.26; SD = 20.27). Moreover, the factor confederate’s age was also significant: *F*(2, 2580) = 43.91, *p* < 0.0001; ƞ^2^_p_ = 0.03. Specifically, IPS was larger with elderly adults (M = 58.92; SD = 20.36) than young adults (M = 56.35; SD = 18.40) and children (M = 54.39; SD = 20.27) and larger with young adults than children (all *p*s < 0.0001). Interestingly, these main effects were qualified by a significant Participant’s Gender × Confederate’s Age interaction: *F*(2, 2580) = 5.73, *p* < 0.003; ƞ^2^_p_ = 0.01. The post hoc test revealed that male participants chose a larger IPS with elderly people (M = 55.94; SD = 18.43) than children (M = 51.78; SD = 18.46) and young adults (M = 52.89; SD = 15.94; all *p*s < 0.001), with no significant difference between children and young adults (*p* = 0.32). Female participants chose a larger IPS with elderly people (M = 61.92; SD = 19.69) followed by young adults (M = 59.81; SD = 18.13) and finally children (M = 57.01; SD = 19.55). All pairwise comparisons were significant (all *p*s < 0.0001; see Figure 3).

Finally, the covariate participant’s age was significant (*F*(1, 1290) = 4.09, *p* < 0.04; ƞ^2^_p_ *p* = 0.01). Nevertheless, all the described effects were still significant.

### 3.3. The Effect of Face-Mask Wearing

The ANCOVA showed a significant main effect of face mask: *F*(1, 1290) = 243.35, *p* < 0.0001; ƞ^2^_p_ = 0.16. Specifically, IPS was shorter with face mask (M = 1.46 m; SD = 0.66) than without (M = 2.49 m; SD = 0.98). In line with previous analysis on the IVAS task, a main effect of participant’s gender appeared: *F*(1, 1290) = 10.83, *p* < 0.001; ƞ^2^_p_ = 0.01, with female participants choosing a larger distance than male participants (M = 2.01; SD = 0.78 vs. M = 1.91; SD = 0.75). Again, even though the covariate participant’s age was significant (*F*(1, 1290) = 31.33, *p* < 0.0001; ƞ^2^_p_ = 0.02), these effects were still significant.

Finally, a Pearson’s correlation analysis showed a strong positive correlation between the two measures of IPS: the mean distance measured by IVAS increased as the preferred distances in meters with and without face mask increased (at least *r* > 0.28 and *p* < 0.0001).

## 4. Discussion

To investigate the relationship between the actual and perceived risk of COVID-19 and the regulation of interpersonal space (IPS), we conducted a multicentric study in six Italian regions with different degrees of COVID-19 infection severity (actual risk). Along with that, we assessed how people perceived the risk of COVID-19 infection (perceived risk) both at the local level (i.e., near where respondents lived) and at the general level and both in the present and in the future. Finally, the impact of respondents’ levels of anxiety was also considered.

The main finding of the present study was that the size of the IPS was not predicted by the actual risk of COVID-19 infection based on objective parameters. Rather, it was predicted by how people perceived the risk of COVID-19 and the related level of anxiety. The proxemics literature has shown that threat is one of the most salient factors in mediating the equilibrium between IPS and social interaction [36,37,38]. However, the extent to which the sense of threat reflects actual danger or personal evaluations has not been clarified. Our results indicate that in IPS regulation mechanisms, the effect exerted by the sense of threat is more related to a subjective evaluation (i.e., perceived risk) than to an objective data-driven evaluation (i.e., actual risk). Actual risk is often contrasted with perceived risk, as the former would be formulated by experts and the latter would reflect an individual feeling. The unfamiliarity of many people with the interpretation of statistical data, combined with how the media represents the data, can lead most individuals to adopt behaviors based on their own representation of risk rather than objective data [39].

The present study suggests that subjective factors may have different weight depending on whether the general or local level of risk is considered. Indeed, the perceived general risk is the factor that drives IPS regulation: the higher the risk perceived by participants in the present at the general level, the larger the IPS. A possible explanation behind this effect can be traced back to an optimistic bias of spatial-geographical nature, as emerged in studies where the perception of environmental problems increased from the local to the global level [40,41]. Consistently, shocking uncontrolled events (e.g., terrorism or earthquake) ([42]; see also [43]) polarize people’s attention toward general risks that are “far” from them [21,22,23,24,44].

The size of the IPS was also influenced by the anxiety generated by the pandemic situation: the more anxious people felt, the larger the IPS. This is compatible with the proxemics literature showing that both trait and state anxiety levels may induce an increase in social distance during potentially threatening situations ([16,17,18,19]; for a review, see [2]). Here we found that during this particular period of pandemic, IPS was influenced by transient psychological reactions toward this adverse situation (state anxiety) rather than trait anxiety, which reflects a stable personality trait. Thus, once again the crucial factor for IPS regulation is how acutely people perceive a threat. Another effect related to the pandemic is that IPS was reduced by about 1 m when other people wore a face mask compared with when they did not. Presumably, wearing face masks allowed people to feel safer and this led them to reduce their interpersonal distance (see also [45]). This reveals a worrying aspect of people’s behavior, as the fact of wearing the face mask does not prevent contagion if it is not also associated with social distancing [46].

As further confirmation that pandemic-related factors influenced participants’ preferred distance from others, the interpersonal distance reported by respondents was dramatically larger (i.e., 54.86 mm) than that reported in a pre-COVID-19 study (i.e., 17.76 mm) that used the IVAS test [12]. With all due caution due to the different size and type of sample, it may be suggestive of a profound change in our social behavior during the pandemic.

Finally, the effects of participant’s gender and confederate’s gender and age demonstrate the reliability of the IVAS as a projective test. Indeed, the present results showed that distance was wider from male than female confederates, and from elderly people than young adults and children. In particular, the increase of the IPS as the age of confederates increased was modulated by the gender of participants: whereas females preferred a smaller distance from children than young adults and from young adults than elderly people, males preferred a similar distance from children and young adults and a larger distance from elderly people (e.g., [2,12,47]). The clear distancing effect with elderly confederates may reflect a higher sensitivity to the risk factor represented by old age in the current pandemic. However, the proxemics literature, using various methods, has reported similar effects of gender and age [2,9,11,12,13,15,48,49,50], making it difficult to disambiguate how much this effect is due to pandemic.

## 5. Conclusions

In sum, the present study revealed that the regulation of IPS was affected by how people subjectively perceived the risk of COVID-19 and the related level of anxiety, not by the actual objective risk. More precisely, it was the general present risk that people perceived regardless of their local context that influenced IPS modulation. This clarifies that the role of threat in prompting avoidant behaviors does not merely reflect environmental events, but rather how the risk is individually perceived and represented. From this perspective, we must consider that the dissemination of information about the pandemic is one of the main means by which we form an idea of the associated risk. Therefore, authorities should communicate news about the pandemic and measures to counter it in a clear and timely manner. More specifically, they should provide citizens with clear information on the dangerousness of the infection, and at the same time they should highlight that it can be controlled by following health instructions [51,52,53]. This could help build a perception of risk that is functional to the adoption of effective mitigation strategies [54]. As regards distance-based mitigation strategies, the effects of age and gender as well as face mask use on IPS regulation could provide useful indications. Males preferred a smaller distance than females and, in general, distance was reduced with children and when other people wore a face mask. These effects should be taken into account when setting up effective press campaigns to promote the maintenance of safe social distances.

From a theoretical point of view, the set of results confirms the basic defensive function of the IPS, showing its deep sensitivity to a threatening social context in which other people are seen as potential virus spreaders. It also shows that the IPS, in order to protect our physical and psychological safety, responds to an early warning that anticipates potential dangers. However, future studies are needed to explore the psychological and contextual factors involved in this protective mechanism.

## Figures and Tables

**Table 1:** The table shows the mean IPS (mm) from the IVAS task, actual risk values and mean perceived risk (PR) values for the present general, present local, future general and future local dimensions and state and trait anxiety as a function of the six Italian regions.

	LOMBARDY M (SD)	EMILIA-ROMAGNA M (SD)	VENETO M (SD)	CAMPANIA M (SD)	APULIA M (SD)	SARDINIA M (SD)
IPS (mm)	57.06 (16.82)	54.09 (15.81)	54.63 (16.78)	57.86 (17.47)	61.19 (19.15)	60.59 (21.09)
Actual Risk	3.83	0.98	0.66	−0.33	−0.37	−0.53
Present General PR	72.81 (19.76)	71.04 (21.26)	71.16 (20.74)	78.32 (19.65)	73.01 (18.86)	74.80 (22.97)
Present Local PR	70.91 (23.56)	54.61 (24.92)	56.02 (23.30)	54.40 (26.49)	52.96 (25.78)	40.01 (25.48)
Future General PR	66.22 (26.20)	62.87 (25.92)	65.36 (24.64)	72.04 (24.31)	73.27 (22.66)	67.52 (27.40)
Future Local PR	65.19 (28.11)	54.29 (27.78)	58.52 (26.25)	63.52 (28.16)	63.02 (27.41)	50.03 (30.49)
State Anxiety	46.87 (11.11)	46.00 (10.82)	45.54 (11.23)	49.46 (11.99)	49.57 (11.48)	45.39 (10.99)
Trait Anxiety	44.17 (9.90)	41.69 (9.55)	43.98 (11.07)	44.67 (11.66)	47.24 (11.56)	42.04 (11.26)

**Table 2:** The table shows the effect of actual and perceived risk (PR) and state and trait anxiety on IPS. *B* coefficients, standard errors, *t* and significance levels for predictors of the outcome variable, standardized beta coefficients and 95% CIs are reported.

Interpersonal Space—IPS										
	*B*	St. Err	*t*	*p*	−95%	+95%	Beta (ß)	St. Err. ß	−95%	+95%
Actual Risk	−0.57	0.34	−1.67	0.09	−1.25	0.09	−0.05	0.03	−0.10	0.01
Present General PR	**0.10**	**0.03**	**3.28**	**0.001**	**0.04**	**0.16**	**0.12**	**0.03**	**0.05**	**0.19**
Present Local PR	0.004	0.03	0.14	0.89	−0.05	0.06	0.006	0.04	−0.07	0.09
Future General PR	0.03	0.03	0.86	0.39	−0.04	0.09	0.04	0.05	−0.05	0.14
Future Local PR	0.06	0.03	1.85	0.06	−0.00	0.13	0.10	0.05	−0.01	0.20
State Anxiety	**0.22**	**0.06**	**3.71**	**0.0002**	**0.11**	**0.34**	**0.14**	**0.04**	**0.07**	**0.22**
Trait Anxiety	−0.03	0.06	−0.5	0.62	−0.15	0.09	−0.02	0.04	−0.09	0.05

## Data Availability

The data presented in this study will be openly available in a publicly accessible repository.
